# Tactical critical thinking program on the tactical efficiency index, declarative and procedural knowledge in male soccer players: a case study

**DOI:** 10.3389/fspor.2024.1469347

**Published:** 2024-12-20

**Authors:** Samuel Jose Gaviria Alzate, Wilder Geovanny Valencia-Sánchez, Frank Esteban Espinal, Jorge Luis Bustamante, Elkin Arias-Arias

**Affiliations:** ^1^ Faculty of Education, University of San Buenaventura-Medellín, Medellín, Colombia; ^2^ Instituto Universitario de Educación Física, Universidad de Antioquia UdeA, Medellín, Colombia

**Keywords:** tactical performance, football, small-sided games, game-based approach, socioconstructivism

## Abstract

**Introduction:**

The purpose of this study was to explore the implications of the in-field implementation of a teaching strategy that promotes critical thinking (TPCT) on tactical ability, declarative, and procedural knowledge. The TPCT is grounded in socio-constructivist theory and aims to enhance players' analytical skills through inquiry, problem-solving, and observation. By emphasizing the socio-constructivist approach, the program fosters skills such as interpretation, analysis, inference, evaluation, explanation, and self-regulation, encouraging players' active participation in questioning and collaborative problem-solving activities. The content of the program focuses on offensive tactical principles of soccer.

**Methods:**

Thirteen male U-14 soccer players (mean age: 13.54 ± 1.00 years; mean soccer-federated experience: 3.92 ± 1.00 years; mean body mass: 39.44 ± 6.09 kg; mean height: 1.61 ± 0.10 m) performed a TPCT intervention strategy over 22 sessions, three times a week. Tactical ability was assessed using the Test for Performance Assessment in Team Sports (PATS), while declarative and procedural knowledge were evaluated with the Tactical Knowledge Test in Soccer (TCTOF). These assessments were administered before and after the intervention.

**Results:**

After the 8-week teaching strategy, there was a significant improvement and substantial increase in the Tactical Efficiency Index (IE) [*t* (12) = 2.61, *p* < 0.05, *r* = 0.73], reflecting a 41% rise from the pretest (M = 0.39, SD = 0.21) to the post-test (M = 0.55, SD = 0.24). Changes in declarative knowledge were minimal, with a 0.31% increase. Similarly, procedural knowledge showed a slight increase (3.53%) that did not reach statistical significance.

**Discussion:**

The findings suggest that integrating critical thinking into sports training could be a strategy to enhance the tactical abilities of young soccer players. While the increase in tactical ability was significant, improvements in declarative and procedural knowledge were minimal. These results highlight the potential of critical thinking-focused programs to impact tactical performance but suggest that further research is needed to explore the broader effects on other types of knowledge.

## Introduction

In team sports, tactics are a complex interplay of analysis, problem-solving, and execution within ever-changing game situations (1). Effective sports performance relies heavily on mental representation and cognitive processing, which translate into on-field decisions and actions (2). Therefore, sports training should prioritize developing players' problem-solving and decision-making skills.

Conventional strategies for teaching sport tactics often rely on replicating standardized game scenarios observed in official matches. For instance, coaches may design drills where players rehearse a specific sequence, such as a pass from the midfield to the right wing followed by a cross to the goal area, typically against passive defenders and with minimal variability in opposition (3). While effective for developing technical precision, these approaches tend to overlook the dynamic and unpredictable nature of real game environments, limiting players' ability to adapt and make strategic decisions in real time (4). To address this limitation, integrating traditional games into tactical development offers a complementary strategy that emphasizes variability, decision-making, and role adaptation. According to Parlebas (5, 6), traditional games are rich in motor interactions, uncertainty, and contextual complexity, which can foster creativity and strategic thinking. These games inherently require players to assume diverse roles, adapt to changing situations, and engage in motor communication, all of which are essential for developing tactical intelligence.

Furthermore, Lavega-Burgués et al. (7) highlight the importance of roles and relationships within traditional games as a means to enhance emotional well-being and interpersonal connections. This relational dimension aligns with socio-constructivist approaches, encouraging players to actively co-construct strategies while navigating the dynamic and interactive challenges presented by traditional games. Such integration not only diversifies tactical training but also enriches players’ ability to transfer learned strategies into formal game contexts.

However, replicating every possible action within the dynamic environment of team sports is unrealistic. Understanding the factors that influence performance, particularly tactical skills, is crucial (4). Success depends on selecting the optimal action in each situation, requiring continuous effective decision-making (8). Caurel and Sánchez (4) highlight the growing interest in understanding the factors that influence performance, especially those related to decision-making tactics. Identifying the elements that translate into highly competitive performance and developing them in practice is essential. Therefore, playing well translates to selecting the most appropriate option in each situation encountered during the game. The ability of a player to consistently make appropriate decisions and execute them through effective technical gestures will determine their performance.

In the dynamic context of sports training, where innovative didactic strategies are constantly emerging, Griffin and Richard (9) underscore the importance of understanding the learning theories that underpin these strategies. Among these, constructivist and ecological theories are particularly prominent.

Constructivism is a learning theory that holds that individuals actively construct their own knowledge from their experiences and interactions with the environment (10). From this perspective, learning is an internal process where individuals assign meaning to new information based on their prior knowledge and mental structures (10). It emphasizes the importance of participation, reflection, and the personal construction of knowledge in the educational process (9–11). On the other hand, socio-constructivism is an extension of constructivism that incorporates the social and cultural dimension in the learning process and knowledge construction (10, 12, 13). This perspective emphasizes the influence of social interactions, cultural practices, and the community environment on the acquisition of knowledge and skills (12). It highlights collaboration, dialogue, negotiation of meanings, and the co-construction of knowledge among individuals within a social and cultural context (9). Socio-constructivism has several pedagogical implications, such as the role of the teacher/coach as a facilitator, the importance of prior learning, the active involvement of learners in the learning process, and the need to allow sufficient time for active knowledge construction (9). By delving into theoretical foundations, teachers and coaches gain relevant and essential information to understand the learning process, make informed decisions about its implementation and potential adaptations, and ultimately predict the outcomes of this process.

Backes et al. (11) propose three prevailing pedagogical principles for applying socio-constructivist theory to the teaching of team sports. The first is associated with facilitating the active construction of knowledge, emphasizing the importance of players actively participating in constructing their own knowledge through exploration and reflection. This principle aims for players to develop mental representations through interaction with information and experiences, allowing them to give personal meaning and coherence to what they have learned. The second principle focuses on facilitating meaningful learning, highlighting the importance of learning having personal meaning for players. This is achieved by involving them in activities that allow them to reflect, evaluate, and critically analyse information, fostering the use of higher-order thinking skills to restructure their prior knowledge. Finally, the third principle relates to facilitating social participation, underscoring the importance of social interaction and collaboration in the learning process. This principle aims to create scenarios that promote cooperation among players, allowing them to work together in problem-solving, decision-making, and developing cognitive skills (11).

Bringing these concepts to the context of team sports, it is possible to infer the benefits provided by teaching strategy based on socio-constructivist theories. On one hand, contextualized learning is highlighted, as learning is conceived in the real scenario (14). More importantly, it is the collective construction of strategies for resolving emerging situations on the playing field that generates greater recognition (13, 15). Being able to resolve from what is collectively built and not from imposed alternatives largely guarantees the potential solution to a situation. Moreover, the opportunity to feel part of the strategy proposition significantly strengthens learning. Backes et al. (11) emphasize that the constructivist perspective has been fundamental in migrating from the traditional paradigm in the teaching-learning process in team sports education. The concept that individuals actively construct knowledge through meaningful interactions between their existing knowledge and new experiences has redefined the roles of coaches and players, established new teaching practices, and enabled innovative approaches like GBA, which prioritize tactical understanding over technical skills in team sports education (9).

Sánchez and Arias (16) compared the learning effects of the Didactic Model of Game Action Competences (DMGAC) with the Didactic Model of Direct Instruction (DMDI) on tactical performance, motivation, and perception of skill in young football players. Their study implemented a program based on a constructivist learning approach, which focused on developing tactical skills in young football players through DMGAC. This constructivist perspective emphasizes the active participation of players in constructing their own knowledge through interaction with the environment and constant feedback. The results showed that participants who received the DMGAC intervention demonstrated significant improvements in tactical performance, intrinsic motivation, and perception of skills compared to those following the DMDI. These findings support the effectiveness of constructivist pedagogical approaches like DMGAC in enhancing the holistic development of young players.

Similarly, the study conducted by Sandy et al. (17) investigated the application of the Teaching Games for Understanding (TGFU) learning model in basketball shooting. This pedagogical approach focuses on introducing students to how they understand sports through the basic concepts of play. In line with the constructivist nature of learning, the study aimed to develop tactical skills in basketball students through the Didactic Model of Game Action Competences (DMGAC). The results showed that applying TGFU-based learning had a significant effect on basketball shooting skills. Participants who received the TGFU intervention demonstrated significant improvements in their tactical performance, intrinsic motivation, and perception of skills compared to those following a DMDI. These findings support the effectiveness of constructivist pedagogical approaches like TGFU in fostering comprehensive growth and skill development in young players.

The concept of Game-Based Approach (GBA) is closely related to these insights. GBA refers to pedagogical models where play and reflection are central to learning. Originating in the 1970s in France and Germany and gaining popularity in the 1980s with Bunker and Thorpe's Teaching Games for Understanding (TGFU) model (18), GBA is rooted in cognitive psychology and ecological perspectives. These perspectives emphasize interaction with the environment and collective contexts as ideal settings for meaningful learning. GBA emerges as an alternative, contextualized learning method through game-associated activities. In this approach, the coach's role shifts to that of a facilitator who promotes dialogue and reflection (18).

GBAs have gained popularity and challenge traditional paradigms in sports teaching, which were based on technical acquisition, migrating to reflective and compression methodologies. These methodologies pose questions about what to do rather than how to do it. Pedagogical practice is seen as an intentional, reflective, and critical teaching-learning process. This intentional teaching demands constant critical reflection during practice. Ultimately, players are expected to understand the game through this reflective exercise (19). Consequently, the development of various skills required in team sports occurs synchronously and in parallel within GBAs. Technical skills, tactical awareness, and decision-making happen simultaneously (20).

Approaching learning from the understanding of the internal logic of team sports involves recognizing how to act in defence or attack to achieve a clear objective. Thus, it is essential to address learning from the tactical principles governing each sport (21). A key component in GBA practices is the role of participants. Firstly, the learner is the central figure of practice. The coach acts as a facilitator guiding the learning process and creating a trusting and safe environment. This learner-centred role generates greater motivation in practice, as players feel like protagonists in their own learning (22, 23).

The constructivist approach explains how social interaction in the game motivates learning. In GBAs, players must decide and accept the consequences of their actions with their group, reinforcing learning through a sense of belonging. An important reason for learning is to create opportunities for players to effectively participate in the game. Therefore, GBAs invite players to constantly analyse their performance, emphasizing cognitive learning from game situations, constructing alternatives based on previous experiences, and creating mental representations. Tactical awareness is worked on from the start, seeking thoughtful players, differing from traditional approaches where technical components are developed first before moving to gameplay. GBA work allows for developing analysis capacity, mental representations, and proposing solutions to faced situations (20).

The nature of GBA highlights the use of modified games to develop technical components in parallel with tactical problems. The coach plays a crucial role in creating challenging situations that force players to make decisions. Small-sided games (SSG) are a consistent alternative for this purpose, involving high-intensity actions and presenting demands similar to competitive games. SSGs are a high-impact pedagogical tool in the physical, technical, and tactical development of players. The different conditioning possibilities allow for working on desired behaviours (24).

Modified games used in GBA must meet two fundamental requirements. Firstly, representation, which relates to maintaining the essence of the real game (20). This representation should match the players' performance level, so the tactical difficulty must be adapted. Secondly, exaggeration, which emphasizes a specific condition demanded by the coach.

Another characteristic of GBA application is constant questioning among participants. Asking questions is used to stimulate participation, thinking, and learning in players. Questions should be tailored to intentions, implicitly inquiring about problems to solve as a team to optimize game performance (21). Gil et al. (25) validated the effectiveness of a comprehensive teaching program based on questioning in decision-making with elementary students in physical education classes. The authors highlight the importance of contextualized practices for learning sports in physical education classes, where understanding the dynamics of sports through questioning is prioritized over merely promoting movement pattern replication.

Characterizing these questions includes assessing declarative knowledge. Firstly, convergent questions correspond to closed questions that invite analysis from prior knowledge, requiring memory of previous experiences, and are regularly used for rule-related topics. On the other hand, divergent questions are open-ended and seek solutions to new situations with equally novel approaches (21).

This questioning exercise leads to verbalization, which supports the knowledge construction process. This verbalization could be guided by incorporating specific recommendations. Firstly, it involves discussing experiences with peers to reach a consensus, known as accurate verbalization. Secondly, it includes judging, which prioritizes and debates the elements evidenced during the game. Thirdly, it encompasses proposing, where alternative solutions are suggested. Additionally, it involves persuading, which focuses on convincing teammates to execute strategies collectively. Finally, it emphasizes conviction, establishing solutions based on solid evidence and recognizing the group's physical and cognitive characteristics (21).

Recommendations for addressing these GBA elements include allowing sufficient time for players to elaborate and propose solutions. Additionally, the quality of questions is crucial to guide intra-group discussions. Sierra-Ríos et al. (26) outline four elements for developing the order of questioning. First, it is essential to establish the activity's objective according to the coach's needs. Second, analysing the players' reality is crucial; this involves recognizing the group's performance level to propose tasks that match their level, a concept known as tactical complexity. Third, it is important to identify obstacles and possible solutions by understanding where group behaviour is hindered and how to resolve it. Finally, establishing an effective plan ensures that these elements are implemented successfully.

Critical thinking is a cornerstone of sports decision-making, involving reflective analysis for reasoned choices in movement tasks (27). Critical thinking can be learned, enabling players to better utilize their cognitive resources. Players can be taught to make decisions more quickly and creatively, spend more time analysing the content or structure of a problem, and be more flexible and willing to modify decisions based on new information (28).

Approaching tactical training from a critical thinking perspective is theoretically advantageous for both coaches and players. This approach seeks to go beyond setting tasks that replicate a real game environment with limited resources. Instead, it aims to develop skills for analysing arguments from an open-minded stance, willing to evaluate and propose solutions based on new information.

Enhancing players' tactical ability requires improving their underlying critical thinking abilities (29). However, much research on tactical ability and decision-making overlooks the critical factors that enable players to solve problems effectively (30, 31). To understand the origin of traditional practices, it is necessary to recall that the teaching of team sports began primarily in physical education classes in the mid-20th century. The pedagogical approaches of the time, focused on gymnastic development, ignored the tactical component and centred on technique. This purely technical focus was carried over into sports development, leaving students unaware of the internal logic of the sport (32). Internal logic refers to the intrinsic structure governing the functioning of motor practices, encompassing the rules, relationships, and dynamics that define their unique characteristics (33, 34). This concept stems from the praxeological model and identifies specific elements that form the internal dynamics of any motor practice: gesture, balancing system, motor space, motor time, motor communication, and motor strategy (35). These elements interconnect to form a coherent system that shapes participants' actions and interactions during the activity.

Building on this, Parlebas (6) highlights that internal logic is pivotal in understanding the universality and diversity of games and sports. It captures the interplay of constraints and possibilities that emerge from the rules and context of a motor practice, which ultimately influence decision-making and behaviour. For instance, in soccer, the internal logic involves spatial-temporal coordination (motor space and motor time), team-based decision-making (motor communication), and strategic adaptation (motor strategy) as players navigate the dynamic uncertainties of the game (33, 35, 36).

Moreover, the variability inherent in motor practices closely ties to motor uncertainty, a central aspect of internal logic. Luchoro-Parrilla et al. (37, 38), emphasize how this uncertainty fosters cognitive engagement, adaptability, and creativity among participants. Similarly, motor interaction, which arises from the relational dynamics between participants, underscores the importance of collaboration and opposition, reinforcing the socio-constructivist underpinnings of training programs like the one explored in this study.

In traditional games, these principles often manifest in culturally specific ways, highlighting the ethnomotor heritage of communities (37, 39). By integrating these elements into tactical teaching strategies, programs can move beyond purely technical instruction to nurture a holistic understanding of the sport's internal logic, enhancing players' tactical awareness and decision-making abilities.

To understand the sequence of events underlying tactics, it is essential to consider that perception is followed by memory and knowledge. One can assert that a player's actions correspond to their interpretation (perception, memory, and decision-making) of the specific situation they face (24). Perception and knowledge act as filters in the decision-making process, as the player must accept or reject the proposed solution before executing it. Therefore, the sequence of events underlying tactics involves perception, memory, and knowledge, which all influence the final decision.

Building on this, in sports, declarative knowledge (knowing what to do) encompasses a player's skill set, while procedural knowledge (knowing when to do what) involves planning solutions in various situations (40–42). The interconnection between these knowledge types is crucial in young players' learning processes, guiding actions, and reducing response times during games (43).

Integrating a critical thinking perspective into tactical training offers theoretical advantages for stakeholders. It goes beyond simply replicating game situations, fostering skills in analysing arguments, proposing solutions, and embracing change (43).

Critical thinking demands the ability to analyse, evaluate, and restructure information to make decisions and act (44). Colln and Giuliano (45) assert that these skills can be integrated into learning processes through exercises and simulations. Indeed, evidence suggests that critical thinking skills can be enhanced through instructional strategies (44, 46, 47). These programs, necessitate a shift in the role of the teacher/coach. From a facilitating stance, they should motivate participants to propose strategies for resolving the situations they encounter. Dwyer et al. (48) emphasize the importance of actively engaging participants in teaching-learning exercises to foster critical thinking skills. This would require teachers to shift away from traditional direct instruction methods.

Fuad et al. (49) maintain that critical thinking skills can be explicitly taught. This learning could be mediated by teachers through the implementation of student-centred instructional models. While the evidence on the effectiveness of explicit critical thinking instruction is not robust, it is important to highlight that it presents the strongest evidence compared to other approaches, such as emergent processes (50). Cosgrove (51) stated that explicitly addressing critical thinking development was superior to the implicit approach. The rationale behind this lies in the fact that the explicit approach provides precise guidance to the learner, allowing them to focus their efforts on resolving a particular situation and reducing the likelihood of confusion regarding the task objective.

Critical thinking relies on both declarative and procedural knowledge, amplifying decision-making effectiveness on the field (52). Therefore, higher levels of both knowledge types are associated with increased critical thinking and players’ ability to make successful decisions (53–55).

Regarding the relationship between GBA and critical thinking, questioning stands out as a knowledge construction tool in tactical problems. Sierra-Ríos et al. (26) mention that the first step in developing critical thinking in players is for them to independently discover solutions to tactical problems presented in game situations. The next step is to invite players to reflect on what happened in the game through questioning strategies. In summary, GBAs are a learner-centred strategy where social interaction and reflective dialogue facilitate learning.

Given this context, there is a significant gap in the research addressing how critical thinking can be systematically integrated into tactical training to enhance players' decision-making skills. This study aims to fill this gap by developing and implementing a critical thinking-based teaching strategy, using GBA as a reference for the tactical training. By focusing on the cognitive processes underlying effective problem-solving and decision-making, this study explores the implications of the in-field implementation of a teaching strategy that promotes critical thinking on tactical ability and procedural and declarative knowledge in male youth soccer players.

## Methods

### Participants

Thirteen male soccer players participated in this study (mean age: 13.54 ± 1.00 years; mean soccer-federated experience: 3.92 ± 1.00 years; mean body mass: 39.44 ± 6.09 kg; mean height: 1.61 ± 0.10 m). All players regularly participated in the club's teaching strategy, attending 3–4 sessions per week (lasting an average of 1.32 ± 0.14 h each) and competing in one official match per week within the regional championship. This corresponds to Tier 2, trained/developmental level, in the Participant Classification Framework (56). Participation criteria included being a U-14 player at the club, having signed informed consent forms from both the player and their parents, and maintaining a minimum attendance of 80% in training sessions over the two months prior to the intervention (adapted from (57). The study excluded players with osteomuscular injuries that could hinder their training or competition. Finally, in 2023, the intervention protocol was evaluated and approved for implementation by the local university's Bioethics Committee.

### Study design

The research used a case study design for the manipulative strategy, incorporating pre- and post-intervention measurements (58). The subjects were intervened with a critical thinking approach for 22 sessions, and evaluated at two moments: pre-test, before the start of the didactic process and pos*t*-test, after finishing the process.

### Measures

This process employed a combination of physical characterization, assessments of tactical principles in soccer, and evaluations of declarative and procedural knowledge. Data collection took place two weeks before the start of the intervention program and immediately at its conclusion. The process relied primarily on ad-hoc surveys for social characterization, alongside previously validated instruments for measuring the dependent variables. Permission to use each evaluation tool was obtained from the respective authors.

### Tactical ability

The validated test PATS by Gréhaigne et al. (59) was used to assess tactical ability. This tool quantifies an individual's overall offensive performance in two key roles: ball management in possession and ball recovery. It achieves this through observation, with established inter-rater reliability exceeding 0.80 and moderate temporal reliability exceeding 0.55 (60). The PATŚs observational methodology aligns with the growing trend in sports science to conduct objective analyses of game actions in competition-like settings (61–63).

### Declarative and procedural knowledge

For this case study with U-14 soccer players, the Offensive Tactical Knowledge Test in Soccer (TCTOF) by Serra-Olivares & García-López (64) was selected to assess declarative and procedural knowledge. This validated questionnaire specifically evaluates tactical knowledge (both declarative and procedural) related to offensive strategies in soccer. The TCTOF demonstrates strong psychometric properties, including high internal consistency (*α* = 0.87), test-retest correlation (*r* = 0.75), and construct and concurrent validity (*p* < 0.01).

### Procedures

Data collection involved video recordings captured using a GoPro Hero 5 digital camera at 120 frames per second during the PATS protocol (59). To ensure the control and quality of the data, one evaluator underwent a comprehensive teaching strategy. This program consisted of theoretical and practical phases. The theoretical phase focused on familiarization with the PATS tool for categorizing soccer game actions as outlined by Gréhaigne et al. (59). The practical phase involved observing game sequences to gain practical experience with the coding process. An evaluator possessed professional backgrounds in physical education and sports as coach in under-14 soccer categories (65).

### Intervention: tactical learning program based on critical thinking (TPCT)

Grounded in socio-constructivist theory, the TPCT aims to enhance players' analytical skills through inquiry, problem-solving, and observation. This design incorporates essential cognitive processes for decision-making, cultivating skills such as interpretation, analysis, inference, evaluation, explanation, and self-regulation (Table 1) (66). By emphasizing the socio-constructivist approach, the program fosters these skills through the active participation of players in questioning and collaborative problem-solving activities. This approach not only strengthens their ability to effectively handle game scenarios but also promotes knowledge construction through meaningful interactions and shared dialogues, in line with the principles of socio-constructivism applied to sports education.

**Table 1:** Tactical efficiency Index (IE), declarative and procedural knowledge of the intervention group.

Variable	Measure (Pretest) Mean, SD Median, IR^a^	Measure (Posttest) Mean, SD Median, IR^a^	%Δ	Confidence interval 95%		*t*/*Z*^a^-value	*p*	ES
IE	0.39, 0.21	0.55, 0.24	41%	−0.29	−0.02	2.61	0.02^b^	*0*.*73*
DK	25.84, 6.00^a^	25.92, 4.21^a^	0.31%	−2.25	1.24	36.5^a^	0.79	*0*.*25*
PK	9.92, 1.58	10.27, 3.53	3.53%	−2.88	2.73	0.64	0.53	*0*.*01*

IE, tactical efficiency index; DK, declarative knowledge; PK, procedural knowledge; M, mean; Mdn, median; SD, standard deviation; IR, interquartile range; %Δ, percentage change; ES, effects size.

Italicized values in Table 1 indicate the specific statistical measures used to analyze non-normal distributions (a, for median and interquartile range) or highlight statistically significant differences (b, for *p* < 0.05).

^a^ Data with non-normal distribution.

^b^ Statistically significant differences between the data (*p* < 0.05).

Implemented within the TPCT framework, the IDEAS model (Identify, Determine, Enumerate, Assess, and Scrutinize) promotes these critical thinking skills.

Aligned with this approach, the TPCT emphasizes explicit learning objectives, openly discussed with the group for at least 15 min prior to practice, adjusting based on group experience. Practice sessions involve small-sided games (SSGs) with specific constraints designed to enhance targeted behaviours or objectives. Each SSG lasts 5 min, and two evaluators per team, ideally players from the same group, observe and quantify actions related to the objectives. Indicators for objective quantification include metrics such as the number of goals scored, successful passes, defensive stops, and transitions. These metrics provide a clear, objective basis for evaluating player performance and progress. Between games, a 5-minute discussion evaluates team performance and proposes strategies, guided by critical thinking questions. This discussion is led by the players themselves, with the coach acting as a facilitator. The aim is to maintain the intensity of the games while ensuring sufficient time for analysis and reflection. The total time for the intervention is approximately 15 min for reflection and 15 min for gameplay (iterative process around at least three rounds). The evaluators, who are players from the same group, do not participate in the initial game but observe and provide feedback during the reflection time. The repetition of the game allows for the integration of recommendations that emerge from the player discussion.

The critical thinking questions include (66):

✓ Is the initial objective being met? (Interpretation/Identification)
✓ What aspects contribute to objective achievement? (Analysis/Determination)
✓ What challenges hinder objective fulfillment? (Inference/Enumeration)
✓ What technical tools could optimize objective attainment? (Evaluation)
✓ What strategies could enhance objective fulfillment? (Explanation/Examination)

**Figure 1:**
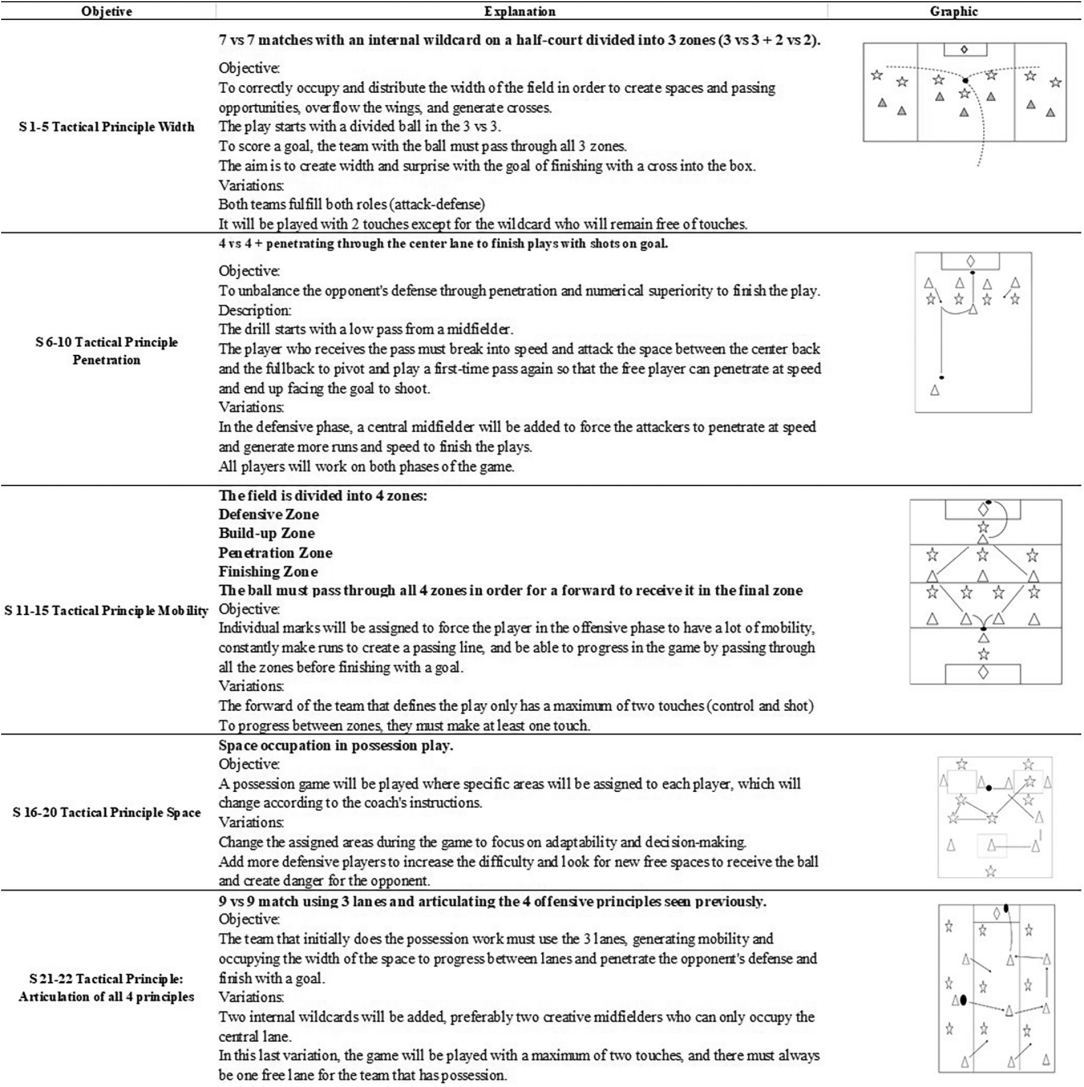
General description of the training sessions for the U-14 soccer team.

Observing technical parameters during gameplay serves to identify the tools needed to address tactical challenges. By intertwining technical and tactical aspects, players can better understand how to apply their skills effectively in game situations, leading to improved performance. Finally, unrestricted play, constituting at least 40% of total practice time, concludes the session, fostering spontaneous application of learned skills. The following is a general description of the training sessions for the U-14 soccer team (Figure 1).

The progression of the games was based on the development of the four offensive tactical principles: width, penetration, mobility, and space (67). The sessions were divided into microcycles of five sessions each, with a specific focus on each tactical principle and a hybrid approach in the last 2 sessions, where tasks aimed at developing all principles were worked on.

Each training session began with a 15-min discussion by the coach on the offensive tactical principle to be addressed. In the initial 15-min discussion, each principle was explained, conceptualized, illustrated graphically, and demonstrated with examples involving some players while the rest observed. This ensured that the players had a clear understanding of the tactical principle being addressed and how it applied to the game. The coach provided verbal explanations, using diagrams to enhance comprehension, and then demonstrate the principle in action on the field with selected players. This hands-on approach helped to reinforce the concepts and allowed players to see how the tactical principle could be applied in real-game situations. Additionally, observing the demonstration allowed the players to visualize their roles and responsibilities within the context of the principle being discussed, fostering a deeper understanding and engagement with the training session (65). Then, a specific warm-up routine was conducted for 15 min, including joint mobility and ball control exercises (rondos). Subsequently, a main task lasting 30 min (Figure 1) was carried out; within the task, there were 5-min reflection breaks every 5 min of task execution. During the reflection time, the coach posed the 5 questions of the TPCT that remained constant throughout all sessions. These questions aimed to guide the players' reflection on their performance, build strategies, or solutions to game problems, all with the aim of raising awareness among the players about the game. Discussions among the players established the strategy, adapted to their needs and qualities, to ensure that the objectives of each session were met. Finally, the practice ended with a game similar to the real game lasting approximately 35 min, with breaks every 10 min to ensure the intensity of the game and spaces for reflection among the players.

Considering there were 13 players on a field roughly the size of a half soccer field (exact measurements unknown because they change in each task), it can estimate an average of approximately 200 square meters per player.

### Bias control

The video analyst, who was selected based on his experience and expertise, underwent an extensive teaching strategy led by seasoned professionals in the field of sports analysis. This training spanned over two weeks and covered the testing methodology in detail. The analyst received comprehensive instructions on the criteria for player selection and the importance of maintaining strict adherence them. To ensure the reliability of his observations, the training included practical sessions where the analyst practiced coding data and counting actions under supervision. Pilot tests were also conducted to evaluate the use and positioning of the camera and to identify and address any potential issues in the data collection process. This thorough preparation was crucial in optimising the accuracy and consistency of the data collected.

### Statistical analysis

The Shapiro-Wilk test was employed to assess data normality in continuous variables. For variables exhibiting a normal distribution, descriptive statistics included means and standard deviations. Conversely, medians and interquartile ranges were presented for variables with non-normal distributions. The dependent student's *t*-test for data with normal distribution and the *Wilcoxon* signed-rank test for data with non-normal distribution were used to contrast the hypothesis testing between performance pretest and posttest of the same group (68). Percentage changes were calculated as [(posttraining value − pretraining value)/pretraining value] × 100. Effect size calculations were performed to evaluate the magnitude of differences in both parametric and non-parametric analyses, following the recommendations adapted from Rosnow and Rosenthal (69). Specifically, Cohen's *d* was used for data with normal distribution, and rank biserial correlation was used for non-normal data. According to Cohen's conventions, an effect size (Cohen's *d*) of 0.2 indicates a small effect, 0.5 indicates a medium effect, and 0.8 or higher indicates a large effect (70). For the rank biserial correlation, values between 0 and 0.1 are considered very small, 0.1 to 0.3 small, 0.3 to 0.5 medium, and above 0.5 large (71). To explore potential correlations between Tactical Efficiency Index (IE), declarative, and procedural knowledge. Pearson correlation tests were conducted for normally distributed variables and Spearman's correlation coefficients for non-normally distributed variables. According to Cohen (70), correlation coefficients (*r*) can be interpreted as follows: values between 0.10 and 0.29 indicate a small correlation, values between 0.30 and 0.49 indicate a medium correlation, and values of 0.50 and above indicate a large correlation. For Spearman's correlation coefficients ($r_{s}$), similar thresholds are applied for interpretation (72). All statistical tests were conducted using Jamovi version 2.3.28, with a significance level set at *α* ≤ 0.05 (*p* ≤ 0.05) and a 95% confidence interval (73). Data visualizations were created using the Python programming language within Visual Studio Code version 1.891.

### Reliability

A highly trained observer with a background in physical education and six years of experience as a soccer coach was involved in the process. The lead researcher provided the observer with a two-week training program to ensure a consistent and standardised approach to evaluating the video recordings. In addition to the training, pilot tests were conducted to assess and guarantee the observer's proficiency in applying the observation criteria. After these preparatory steps, the pre-test and pos*t*-test videos were reviewed a second time, 21 days later (74). The total actions were analysed, considering an amount higher than recommended literature (75). Cohen's Kappa score was calculated to be 0.89, which reflects substantial and nearly perfect intra-observer reliability, confirming the consistency of the observations over time (76).

## Results

Thirteen male U-14 soccer players (mean age: 13.54 ± 1.00 years; mean soccer-federated experience: 3.92 ± 1.00 years; mean body mass: 39.44 ± 6.09 kg; mean height: 1.61 ± 0.10 m) performed a TPCT intervention program over 22 sessions, three times a week. A total of 683 tactical actions were assessed using PATS in both the pre-test and pos*t*-test phases. In Table 1 there are the results of the IE, Declarative and Procedural Knowledge of the intervention group.

**Figure 2:**
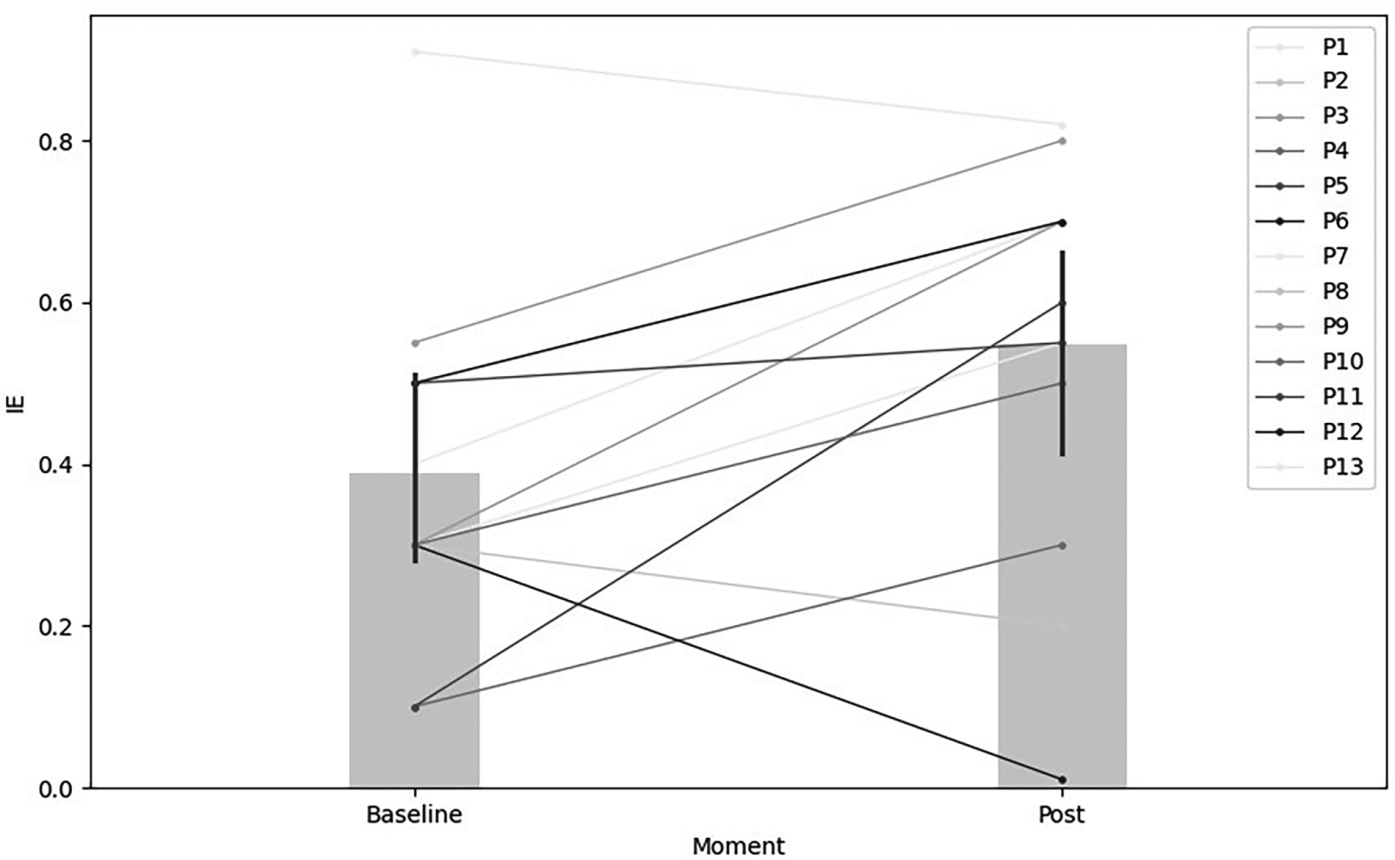
Statistical comparison of IE between pre-test and post-test after 22 TPCT sessions.

Regarding IE, a significant statistical increase after 22 TPCT sessions [*t* (12) = 2.61, *p* < 0.05, *r* = 0.73, %Δ = 41]. This reflected a 41% rise between the pre-test (M = 0.39, SD = 0.21) and the post-test (M = 0.55, SD = 0.24). These results are illustrated in Figure 2.

### Declarative knowledge

**Figure 3:**
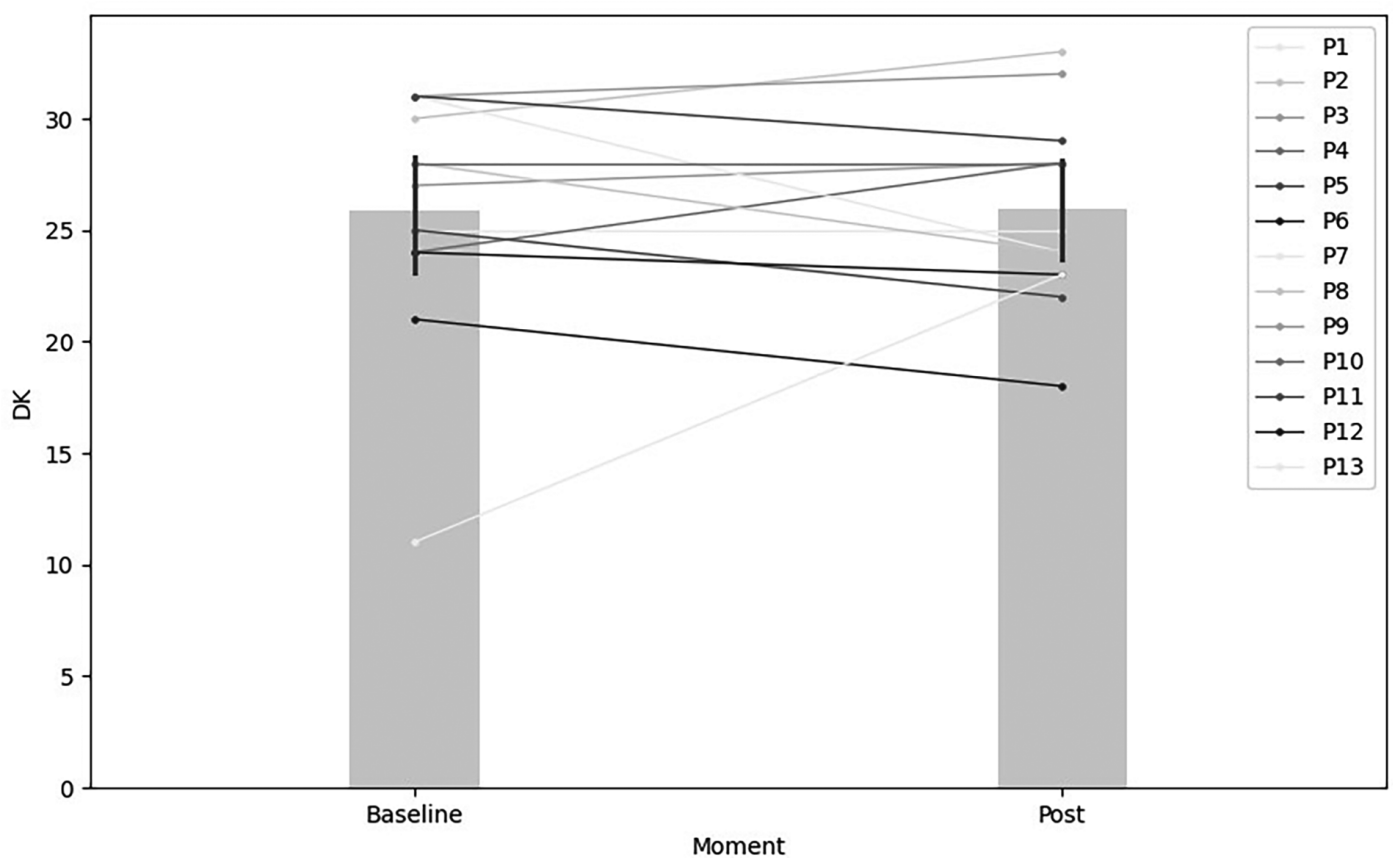
Declarative knowledge comparison between pre-test and post-test after 22 TPCT sessions focused on offensive tactical principles.

Concerning declarative knowledge, there was a negligible 0.31% increase from pre-test (*Mdn* = 25.84, IR = 6.00) to pos*t*-test (*Mdn* = 25.92, IR = 4.21) after 22 TPCT sessions focused on offensive tactical principles. This difference lacked statistical significance, with a small effect size (*Z* = 36.5, *p* > 0.05, *r* = 0.25, %Δ = 0.31). These results are illustrated in Figure 3.

### Procedural knowledge

**Figure 4:**
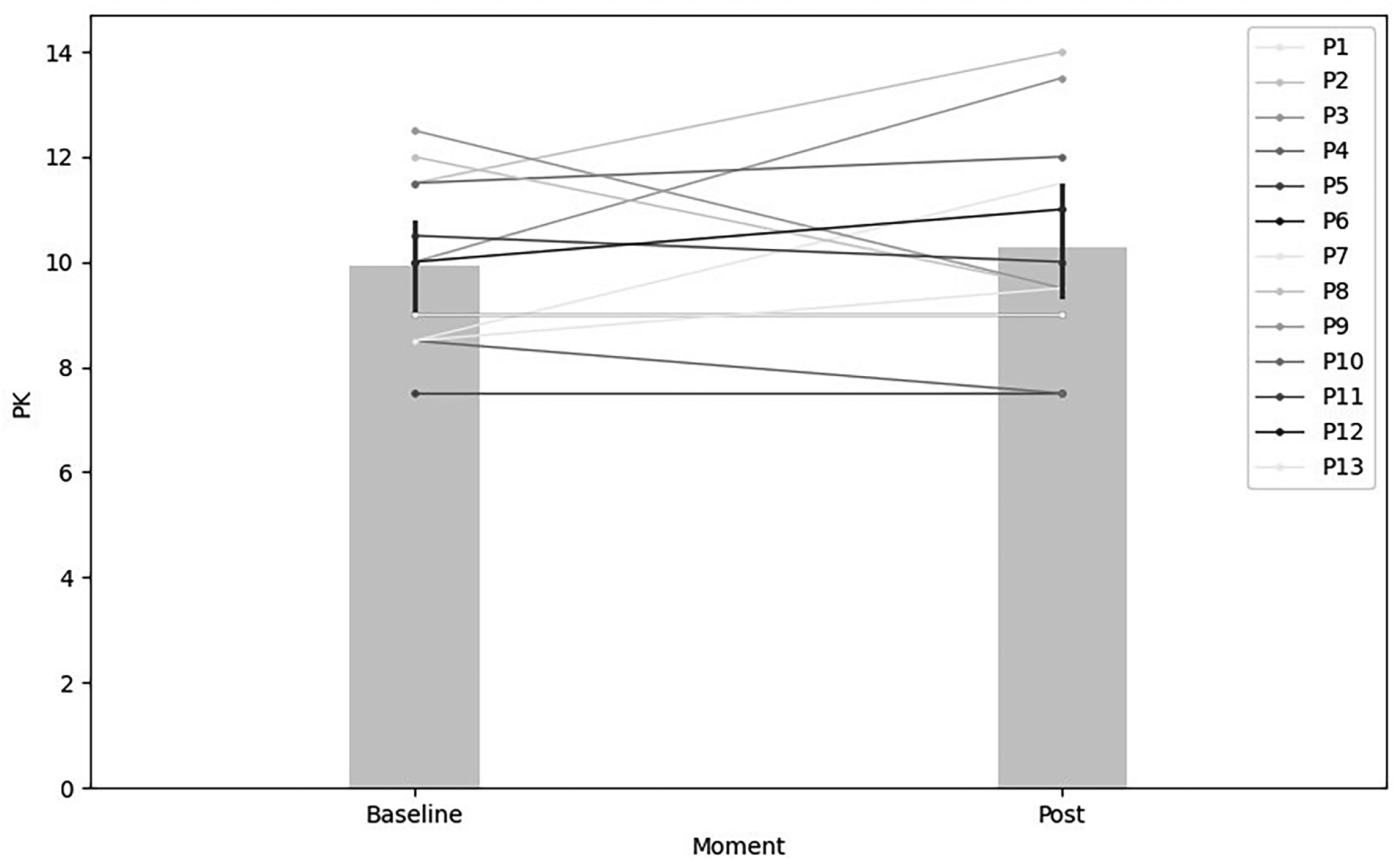
Procedural knowledge comparison between pre-test and post-test after 22 TPCT sessions focused on offensive tactical principles.

Regarding procedural knowledge, the group exhibited a 3.53% increase from pre-test (M = 9.92 SD = 1.58) to pos*t*-test (M = 10.27, SD = 3.53). However, this change did not reach statistical significance, accompanied by a small effect size [*t* (12) = 0.64, *p* > 0.05, *r* = 0.01, %Δ = 3.53]. These results are illustrated in Figure 4.

No significant correlations were observed at the *p* > 0.05 (one-tailed) level between post-test measures of the tactical efficiency index, declarative knowledge, and procedural knowledge (IE-DKpost: *r* = −0.22; IE-PKpost: *r* = −0.12; PK-DKpost: *r* = 0.38).

## Discussion

This study explores the implications of the in-field implementation of a teaching strategy that promotes critical thinking on tactical ability and procedural and declarative knowledge among U-14 soccer players. The study measured changes in tactical efficiency indices (IE), declarative knowledge, and procedural knowledge. In general, the study suggests that the TPCT program may have contributed to improvements in the tactical efficiency index of young soccer players, with a more notable effect on procedural knowledge (3.53% increase) compared to declarative knowledge (0.31% increase). Despite the lack of significant correlations between the tactical efficiency index, declarative, and procedural knowledge, the program's emphasis on understanding game principles and strategies, along with promoting critical thinking and adaptability, underscores its potential in developing both cognitive and tactical skills in youth soccer players.

### Tactical performance

Regarding the IE, a crucial determinant of game success, defined as the relationship between offensive factors such as balls recovered in attack, successful shots on goal, and ball losses (59), our findings align with previous research. A systematic review by Silva et al. (77) investigating the impact of teaching strategy on decision-making in young team sports players found that cognitive-based strategies with collective discussions yielded superior improvements compared to strategies without them. This is particularly relevant to our study, where TPCT participants demonstrated significantly higher tactical performance indices. This suggests that the TPCT program, with its emphasis on critical thinking and collaborative learning, may have contributed to improved tactical decision-making in the participating players.

The effectiveness of TPCT likely stems from its multifaceted approach (78). By fostering analytical skills and collective problem-solving (77), TPCT equips players to make better tactical decisions on the field. Additionally, the program's contextualized transfer approach ensures skills are developed based on players' input and collective understanding, facilitating a smooth transition to game situations. Furthermore, TPCT emphasizes a deeper grasp of game principles and strategies, empowering players to make informed tactical choices. The program also cultivates critical thinking, encouraging players to find innovative solutions tailored to their strengths and adapt their thinking in dynamic game situations. Finally, TPCT tasks players with recognizing and responding to changing contexts, enhancing their adaptability and ultimately improving their overall tactical performance.

Griffin and Richard (9) identify two key principles in socio-constructivist theory that underpin the effectiveness of strategies like TPCT: prior learning conditions new learning, and learning is an active exercise where learners negotiate their understanding based on their experiences. “Negotiation” in this context refers to the collective questioning exercise that occurs constantly. These key principles determine three fundamental considerations, according to Griffin and Richard: reality, constructed through human activity; knowledge, which is fostered when learners make sense of something through interaction with each other and their environment; and learning, which is considered meaningful when the learner engages in social action, such as interactions or collaborations. In conclusion, coaches can benefit from incorporating cooperative learning strategies, dyadic interactions, and idea debate into their training sessions to enhance the performance and learning experience of players in socio-constructivist environments. Furthermore, Fagundes et al. (79) found that comprehension models, akin to TPCT, positively influence cognitive dimensions in racket sports, aligning with our study's outcomes. TPCT's focus on optimizing the player-environment relationship and involving players in strategy formulation may help facilitate more effective cognitive and tactical skill development. Additionally, task constraints within TPCT sessions prompt decision-making and deepen players' understanding of game implications, leading to more meaningful learning.

The nature of TPCT tasks, conducted in confined spaces with specific constraints, fosters the development of cognitive and coordinative skills such as concentration, attention, and anticipation, as supported by research (80–82). Idarraga and Valencia-Sánchez (57) note that practice spaces emphasizing cognitive and coordinative elements induce variability and uncertainty, prompting players to resolve cognitive conflicts and hone their skills. This ecological complexity shared by psychokinetic games and TPCT tasks likely underlies the observed tactical performance improvements.

Restrictions integrated into training practices, as emphasized by Gonzalez-Artetxe et al. (83), enhance task complexity and spur players to explore diverse strategies. Specific constraints, such as ball-touch requirements before a shot on goal, heighten concentration and precision, fostering player commitment and behaviour internalization. Overall, the structured framework provided by restriction-oriented training practices augments tactical performance in young soccer players.

### Declarative and procedural knowledge

The present study delves into both declarative and procedural knowledge within the context of soccer. Declarative knowledge refers to players' comprehension of game skills, essentially understanding “what to do” in various game scenarios (40). On the other hand, procedural knowledge entails the ability to devise effective solutions and execute them appropriately during gameplay (40).

In assessing these dimensions, we made a deliberate decision not to directly measure critical thinking skills, despite the TPCT's focus on fostering such abilities. This decision was motivated by several factors. Firstly, utilizing lengthy standardized tests such as the IMPC OSBA VS2, as proposed by Salazar-Blandón & Ospina-Rave (84) and validated for Colombians, could impose undue burdens on young players. These tests have the potential to distract from their focus and performance within the soccer context (64). Secondly, these tests may not be tailored specifically for sports contexts, potentially yielding results that inadequately capture the critical thinking skills pertinent to soccer (85, 86).

Distinguishing between declarative and procedural knowledge is crucial for understanding their roles in soccer players’ tactical decision-making. Declarative knowledge, knowing what, entails expressing knowledge about a task, while procedural knowledge, knowing how, involves skilful task execution (87). Previous studies link high procedural knowledge levels to high declarative knowledge levels (8, 88, 89).

TPCT prioritizes practical skill application in real-game scenarios (procedural knowledge) over theoretical knowledge acquisition (declarative knowledge). While TPCT's initial clarifications aim to foster game logic understanding and reflection, they may not suffice for significant declarative knowledge gains in under-14 soccer players. Hence, the higher increase in procedural (3.53%) over declarative knowledge (0.31%) in our study.

These findings echo Gamero et al.'s (40) quasi-experiment on basketball students aged 11–12, analysing three teaching methods' effects, including Tactical Game Approach (TGA), on declarative and procedural knowledge. Similar to TPCT, TGA emphasizes game-based teaching and player involvement in tactical improvement through cognitive engagement and questioning (40).

TPCT's nature, promoting player participation, understanding, and reflection, suggests its efficacy in developing declarative and procedural knowledge compared to rigid, coach-centred approaches. Though our study lacked a control group, previous research (30, 40, 90–92) supports this notion.

The absence of significant correlations between the tactical efficiency index, declarative, and procedural knowledge doesn't negate their relationship. Rink et al. (93) suggests substantial cognitive changes in sports contexts require 15–18 weeks of instruction, highlighting the need for further research to elucidate these relationships in young soccer players within longer study durations.

Beyond the quantitative results obtained, the implementation of the TPCT program stood out due to the active participation and acceptance of the players, who fully engaged in the proposed dynamics. This level of commitment was evident in the players' willingness to tackle the inherent challenges of the program, such as adapting to tasks centred on specific tactical principles and critically reflecting on their tactical decisions. Although this perception was not explicitly measured, the researchers observed that the TPCT design, grounded in the co-construction of knowledge and collective problem-solving, fostered a collaborative and motivating learning environment. This approach, while not guaranteeing performance improvements, was positively received by the participants, who demonstrated a high level of engagement with the program's activities. The focus on specific tactical principles and collective problem-solving fostered a positive environment of participation and discussion among the players. Although the study's limitations prevent definitive conclusions about the program's impact on performance, the observed acceptance and willingness of players to engage with the TPCT approach suggest its potential as a valuable framework for promoting critical thinking and adaptability in youth sports training.

Thus, the rapid evolution of sports performance continues to challenge coaches and players to develop effective training processes. Traditionally, the focus has been on individual attributes such as technical or physical skills. However, in team sports, success crucially depends on coordination within a dynamic environment. Players must adapt and solve problems in real-time, highlighting the need for a holistic approach that addresses both individual and collective coordination.

The socio-constructivist theory provides a robust conceptual framework for understanding how teams construct knowledge through interaction with their environment. In team sports, players are constantly seeking to develop solutions to the challenges presented by the game, their teammates, and their opponents. This requires continuous reorganization at both individual and group levels to achieve competitive success.

### Limitations

The kind of the design study does not allow establishing relations of cause and effect between independent variables and dependent variables because single-case design does not have a control group, and this absence of a control group limits the ability to attribute observed changes solely to the intervention (58). It is possible that external factors contributed to the results. Furthermore, this result is applied to the participants because the study did not make sample calculations, and the participants did not represent the population, and the participants did not choose the random and representative of the broader Colombian U-14 soccer player population.

Another limitation is related to the tool used in this study. The tools employed to evaluate the intervention's outcomes were not specifically designed to measure critical thinking, and the survey TCTOF was not to make cross-cultural adaptations in the Colombian context (61).

The results offer the relationship between declarative knowledge, procedural knowledge, and tactical efficiency; they do not capture the multidimensional nature of critical thinking. Two key factors drove the decision to measure declarative and procedural knowledge instead of directly measuring critical thinking. First, existing critical thinking tests are often lengthy and may be cognitively demanding for U-14 players, potentially impacting their focus and performance (86). Second, the tests available for the Colombian context (84) did not specifically design for soccer players, potentially compromising the validity of results by neglecting to address critical thinking skills pertinent to the sport. This underscores the need for the development of tests specifically tailored to assess critical thinking in sports contexts, enabling more accurate and representative analyses (86). Despite these limitations, the study holds significant theoretical and practical insights.

## Conclusion

The findings could suggest that integrating critical thinking into sports training can be a strategy to enhance the tactical abilities of young soccer players. TPCT emerges as an innovative teaching strategy with the potential to enhance tactical performance in soccer by empowering players to make efficient decisions on the field through the development of cognitive skills, particularly critical thinking. This approach could enable players to process and analyse information effectively, optimizing their tactical execution. While this study provides valuable insights into the application of TPCT and generates hypotheses for future research, further experimental studies are necessary to validate and generalize these findings to youth male soccer players. Additionally, this work offers practical guidance for coaches working with similar age categories, representing an incremental step in advancing tactical training methodologies in football.

### Practical implications

The study's findings carry significant practical implications for soccer, underscoring the importance of strategies aimed at refining analytical and problem-solving skills within sports settings (94). It is crucial for all stakeholders, not just coaches, including team managers, sports psychologists, and youth development coordinators, to recognize the value of investing in the development of underlying tactical skills, such as critical thinking. By doing so, they can contribute to the holistic growth and performance enhancement of young soccer players.

TPCT's lies in players' active involvement during training, where they continuously propose solutions and self-assess, fostering cognitive skill acquisition and tactical development. The culture of critical thinking promoted by TPCT enriches training processes and enhances players' tactical performance, with less coach intervention.

## Data Availability

The raw data supporting the conclusions of this article will be made available by the authors, without undue reservation.
